# Real-world utilization of Cenobamate as adjunct therapy in office-based neurology: practical tips and insights for titration

**DOI:** 10.3389/fneur.2025.1558614

**Published:** 2025-06-13

**Authors:** Patrick M. House, Lars Wiese

**Affiliations:** ^1^Epileptologicum Hamburg, Hamburg, Germany; ^2^Zentrum für Neurologie in Berlin-Charlottenburg, Berlin, Germany

**Keywords:** Cenobamate, refractory epilepsy, seizure freedom, antiseizure medication, neurologist, epileptologist, epilepsy care pathways, outpatients

## Abstract

**Introduction:**

Epilepsy poses significant management challenges, particularly in patients with refractory epilepsy where conventional antiseizure medications (ASMs) are ineffective. Cenobamate (CNB), a recently approved third-generation ASM, has shown unprecedented efficacy as an adjunctive therapy in clinic-based practice. However, to date, its use by office-based neurologists in Germany remains relatively limited. One reason for this is its perceived complexity and false perception as a medication of last resort. This study focuses on the logistics of German care pathways, CNB titration, and ASM combinations in a first cohort of office-based outpatients. It also gives a glimpse into which ASMs are being used in the office-based setting in comparison to population and clinic-based data sources.

**Methods:**

The cohort comprised 55 patients from two office-based outpatient practices (*Niedergelassene*) in Berlin (*n* = 25) and Hamburg (*n* = 30). All patients had a history of refractory epilepsy despite optimal treatment with existing ASMs. Patients were initiated on CNB from the month of approval (June 2021) to March 2023. Data on prior ASM usage were collated alongside clinical data, which included seizure frequency and drug load reduction outcomes to March 2025.

**Results:**

Prior to CNB initiation, patients at both office-based practices had similar levels of 1–2 concurrent ASMs (Berlin 80%; Hamburg 77%). The most common ASMs were voltage-gated sodium channel blockers (VGSC), Levetiracetam (LEV)/Brivaracetam (BRV) synaptic vesicle protein 2A (SV2A) inhibitors, and Perampanel (PER). CNB titration was configured into a quarterly office-based outpatient schedule. All patients had seizure reductions in-line with published and real-world evidence, and were compliant.

**Discussion and conclusion:**

CNB is a valuable adjunctive therapy suitable for refractory epilepsy outpatients attending office-based neurologists. A slow titration schedule helped mitigate most side effects. Despite differences to clinic-based practice, in office-based outpatient practice CNB can be broadly used. It can be prescribed to patients on conventional therapy who are still having seizures and have failed two or more other ASMs. By reporting experiences of CNB titration, seizure, and drug load reduction outcomes in office-based neurology, this study will give German office-based outpatient neurologists evidence to support both CNB and other third-generation ASM use in their practice.

## 1 Introduction

Epilepsy, a neurological disorder characterized by recurrent seizures, affects around 50 million people globally and an estimated 600,000 in Germany alone (1–3). Across the world, anti-seizure medications (ASM) are the main treatment option (2). A recent evaluation of epilepsy treatment trends in Germany showed that ASM prescription patterns are shifting toward newer, third-generation drugs (4). This shift is attributed to their ease of use, more tolerable side effects, good response rates, and reduced safety concerns when compared with older ASMs. Yet, epileptic seizures continue to pose significant management challenges. Approximately one-third of epilepsy patients experience drug-resistant epilepsy, even after trying three or more ASMs (1, 5–10). In Germany, recent publications and IQVIA™ data suggest that there are a high number of patients with inadequate ASM regimens and refractory epilepsy (6, 11, 12), with emergency admissions of patients with known seizure disorders estimated at a rate of 135.4 per 100,000 adult inhabitants (13).

In Germany, epilepsy is a condition in which the bulk of patient care happens as an outpatient, yet the pathway to outpatient epilepsy care presents unique challenges. A typical patient’s pathway often starts with a drop seizure, leading to acute care in one of Germany’s 2,500 hospitals. Most patients then receive a short-term discharge prescription (*Entlassrezept*) (14), and transfer to a general practitioner (GP), epilepsy outpatient department (present in only 28% of hospitals), or office-based outpatient neurologist (*Niedergelassene*) (15, 16). The German Medical Association (*Bundesärztekammer* (BÄK)) reports a total of 9,636 neurologists in Germany, of whom 2,899 are outpatient neurologists (15). Recent research suggests a high number of patients with inadequate ASM regimens and refractory epilepsy (6, 11–13): nationwide there are 216,000 patients who have tried three or more ASMs of which 81,600 have never seen an outpatient neurologist, having received care solely from GPs or hospitals (11). The remaining 62% (134,400) patients are under the care of outpatient practices. These practices include the 54 specialized epilepsy outpatient centers certified by the German Society for Epileptology (*Deutsche Gesellschaft für Epileptologie* (DGfE)) (17, 18) and 35 adult outpatient practices (*Schwerpunktpraxis* (SPP)) (19). The high number of patients means that the most significant patient loads are distributed to office-based outpatient neurologists. Yet not all office-based outpatient neurologists have the capacity or expertise to care for local refractory patients—previously published data show marked regional variations in care (11, 12, 17, 20, 21). There are some large differences in ASM prescription choices between GPs and hospital clinic neurologists. For example, there is a higher use of Lamotrigine (LTG) by neurologists than GPs (27% v 17% for first ASM; 29% v 20% for all ASMs). These differences are likely to be present for office-based outpatient neurologists and indicate the possibility of different treatment regimens. There are 81,600 patients who have not yet seen a neurologist but, based on their IQVIA™ prescription data, are likely to have refractory epilepsy and would benefit from outpatient neurologist care.

Researchers have highlighted the urgent need for increased use of ASMs that increase seizure freedom rates with a balanced tolerability profile, allowing a reduction of concurrent ASMs (22). In light of the German patient data and epilepsy care pathways, the evidence is that German office-based outpatients with refractory epilepsy need treatment from neurologists who can prescribe the most effective and safe ASMs. With newer, third-generation ASMs such as Cenobamate (CNB) demonstrating significant advantages when compared to traditionally prescribed Levetiracetam (LEV), Lacosamide (LCM), and LTG, it is important to provide insights and data to support and give confidence to office-based outpatient neurologists in treating patients with refractory epilepsy.

In June 2021, CNB was approved by the European Medicines Agency (EMA) for use in adult populations (23). This third-generation ASM has shown unprecedented efficacy as an adjunctive therapy in controlling seizures. Additionally, when combined correctly with other ASMs, CNB results in low side effects (12, 24–32). However, even though the majority of CNB patients are treated as outpatients, most published CNB studies reference data from patients seen by neurologists in hospital clinics. Office-based outpatient neurologists and their GP colleagues follow different care pathways and experience distinct challenges compared to their hospital-based colleagues. To provide customized guidance and learning from experience on ASM prescribing, real-world data from studies on CNB used specifically within office-based outpatient neurology practice are required.

To increase current understanding of best practices for combinations of commonly used ASMs for office-based outpatients, the first aim of this study was to create a profile of office-based outpatients by discerning the specific ASM combinations prescribed prior to CNB initiation. Secondly, clinicians’ narrative and qualitative experience was compiled regarding which combination partners for CNB are advantageous from an office-based outpatient perspective. The third aim was to gather and compile data on quarterly CNB titration schedules for use by other office-based outpatient neurologists.

## 2 Methods

The cohort comprised 55 patients from two office-based neurologists in Germany (*n* = 25 and *n* = 30). The patients were sourced from two distinct cities—Berlin and Hamburg—each with a population exceeding one million. At the time of data assessment, these subjects constituted the entire population of consecutively treated patients who had been prescribed CNB in the respective practices during the specified timeframe.

Anonymized patient data were assessed for the period June 2021 to June 2023 (25 months). Follow up seizure data were also collected in March 2025. All patients had a history of drug refractory epilepsy despite optimal treatment with existing ASMs and were initiated on CNB as adjunctive therapy. Of the 55 patients, 47 (85%) were treated on-label per German regulations (33). The eight patients treated off-label were administered doses over 400 mg/d. Anonymized clinical data collected included age (years/months), sex, epilepsy etiology and specific diagnosis, cognitive function, seizure frequency, number and type of ASMs received prior to and after CNB initiation, CNB start date and titration details, side effects and adverse events, and outcome by seizure reduction (seizure reduction <50%; seizure reduction >50%; seizure freedom; seizure increase; no change). Efficacy and tolerability of CNB was assessed by the neurologists through quarterly in-person appointments, reviewing side effects and adjusting ASM doses accordingly. All patients (except one Berlin patient) underwent laboratory and cardiac investigations at each appointment [including plasmatic assessment of ASM, hepatic function, sodium levels, electroencephalogram (EEG) registration, and electrocardiogram (ECG)].

In addition, the observations from each site were compiled and a consensus was reached regarding the clinical reasoning process around optimal CNB prescriptions, with a focus on the logistics of titration and ASM use.

## 3 Results

### 3.1 Cohort demographics

**Figure 1:**
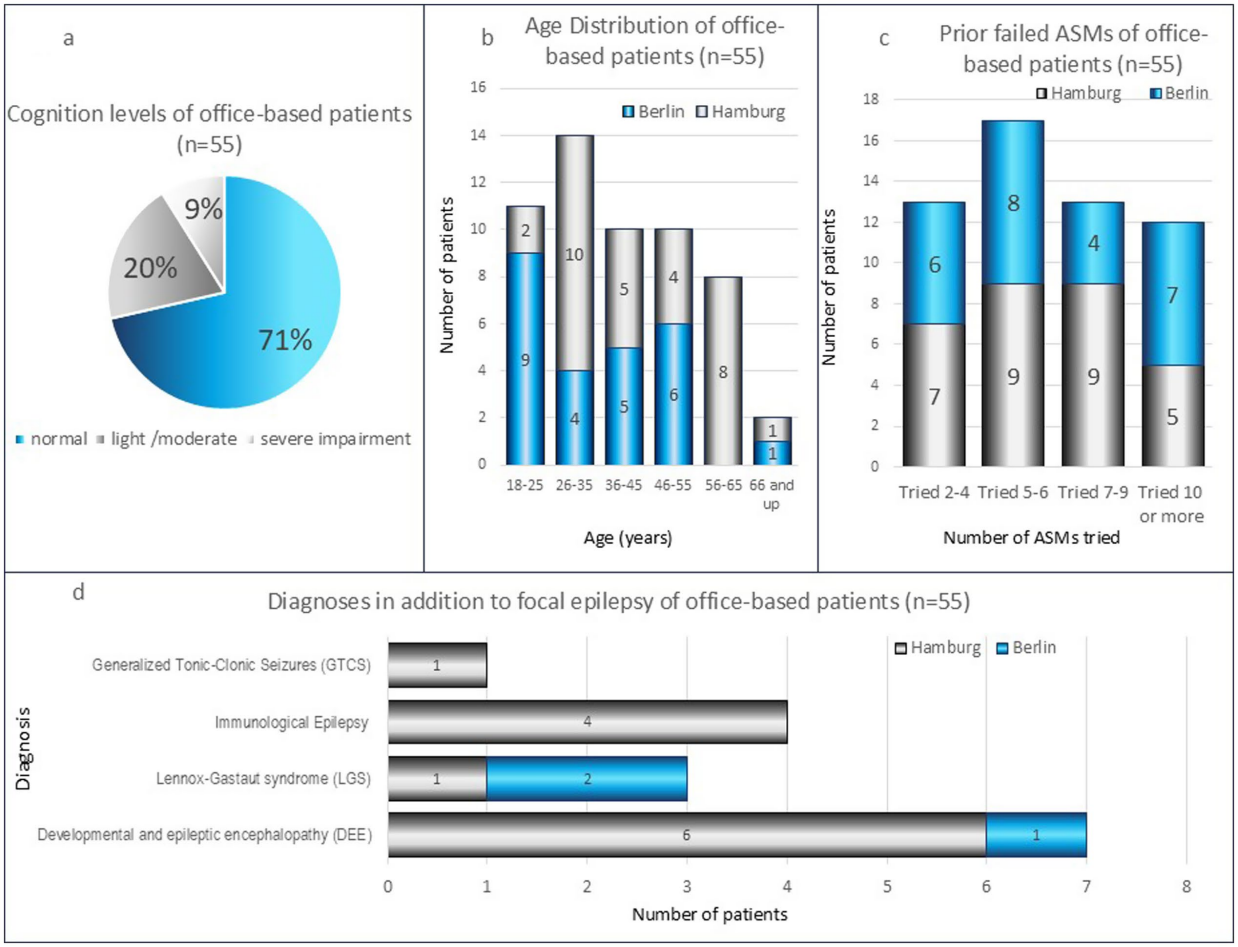
Demographic information for the office-based outpatient cohort, n=55. **(a)** Spectrum of cognitive abilities reflected diversity within the studied population; **(b)** age distribution was heterogenous; **(c)** patients had taken between 3 and 18 failed ASMs or ASM combinations (mean=7.4) prior to CNB initiation; **(d)** all patients were diagnosed with focal epilepsy but some patients had more than one additional diagnosis. Although LGS is a DEE, they are shown separated in the figure. ASM, anti-seizure medication; CNB, Cenobamate; n, number of patients; LGS, Lennox-Gastaut syndrome; DEE, developmental and epileptic encephalopathy.

When compared with demographic data from IQVIA™ (11, 12), the sample is essentially representative of the German epilepsy population (Figure 1). The spectrum of cognitive capabilities reflected significant diversity within the studied patient population (Figure 1a). Furthermore, the sample represented patients from different age groups, thereby ensuring heterogeneity (Figure 1b). At the time of CNB initiation, duration of disease ranged from 1 to 58 years (mean = 25 months) and patients had taken between 3 and 18 failed ASMs or ASM combinations (mean = 7.4, Figure 1c). All patients had a diagnosis of focal epilepsy, and 11 additionally had developmental and epileptic encephalopathy (DEE), Lennox–Gastaut syndrome (LGS), immunological epilepsy, or generalized tonic–clonic seizures alone (GTCS) (Figure 1d). For further details please see Supplementary Table S1.

### 3.2 Cenobamate dosage and pharmacological burden

**Figure 2:**
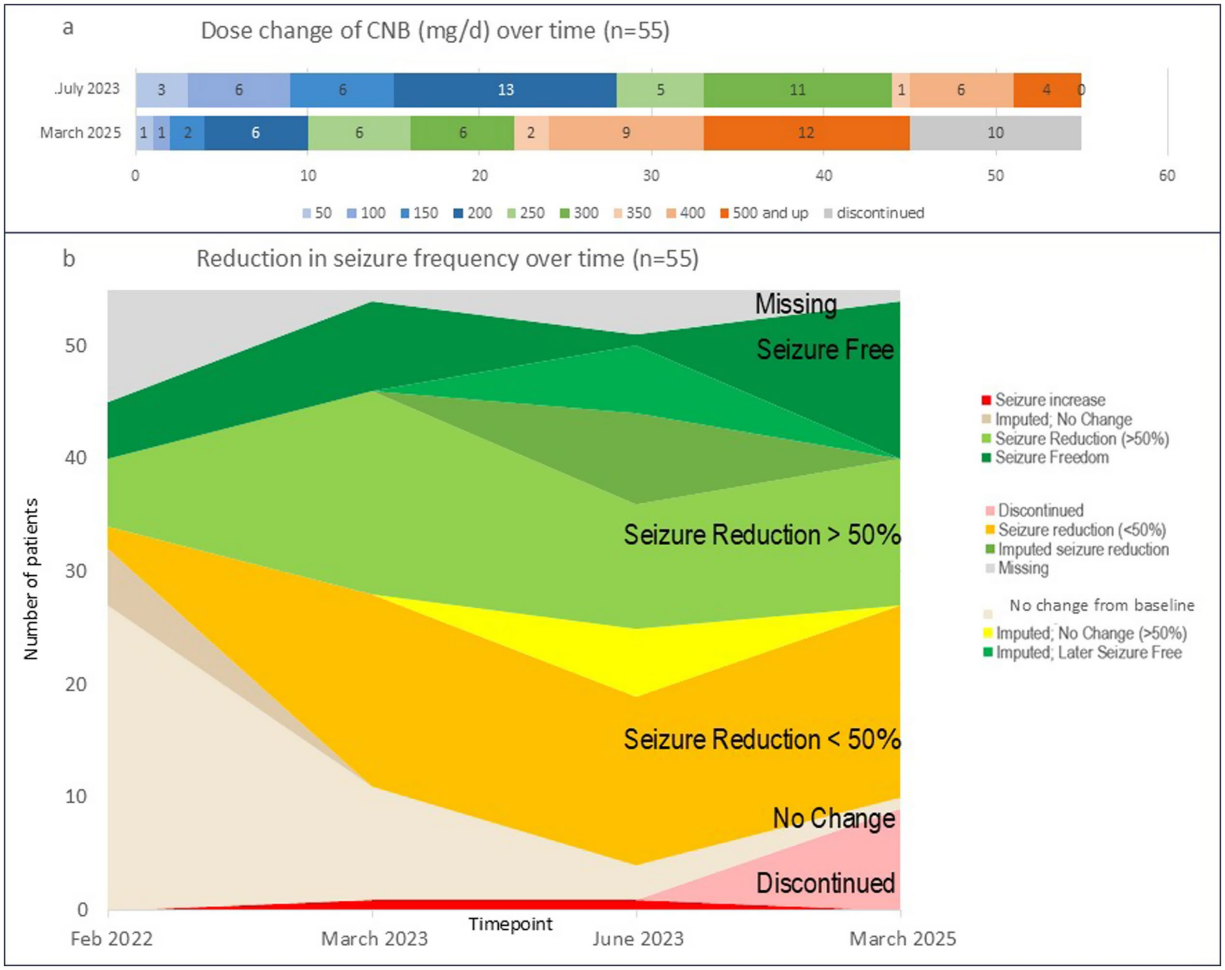
Changes in CNB dose and seizure frequency over time for the office-based outpatient cohort, n=55. **(a)** All patients initiated CNB with 12.5 mg/d and over time dose was increased until positive seizure outcome or discontinuation; **(b)** seizure reduction across the whole cohort at timepoints February 2022, March 2023, June 2023, and March 2025. Green represents patients who were seizure-free or had a seizure reduction >50%. Orange represents patients with seizure reduction <50%, beige represents patients with no change. By March 2025 more than half of patients were seizure-free or had >50% reduction. CNB, Cenobamate; mg/d, milligram per day; n, number of patients.

All patients started Cenobamate on dosage of 12.5 mg/d. Published European titration schemes were followed (33). Effective end dose was determined by seizure freedom or seizure reduction balanced with side effect tolerability. Figure 2a shows dose changes from July 2023 to March 2025. Detailed information is available in Supplementary Table S1. Colors represent dosages (e.g., 50, 150, 300 mg/d) over this time period, patients increased their dose until reaching their effective end dose or discontinuing.

For all patients the pharmacological burden of concurrent ASMs was reduced. Detailed information is available in Supplementary Table S1. Reduction was always due to a combination of responding to side effects and a proactive approach to reduce pharmacological burden.

#### 3.2.1 Seizure reduction outcomes

All patients had seizure reductions in-line with published and real-world evidence. At the end point of the CNB initiation window (June 2023, 3–24 months after CNB initiation, mean = 9.5 months), no patients had dropped use of CNB. Seizure outcomes data were also collected in March 2025. Figure 2b shows seizure reduction across the whole cohort at respective timepoints February 2022, March 2023, June 2023, and March 2025. Green represents patients who were seizure-free or had a seizure reduction >50%. Orange represents patients with seizure reduction <50%. Beige represents patients with no change. By March 2025 more than half of patients were seizure-free or had >50% reduction.

### 3.3 ASMs prior to CNB initiation

#### 3.3.1 Prior ASMs by medication

**Figure 3:**
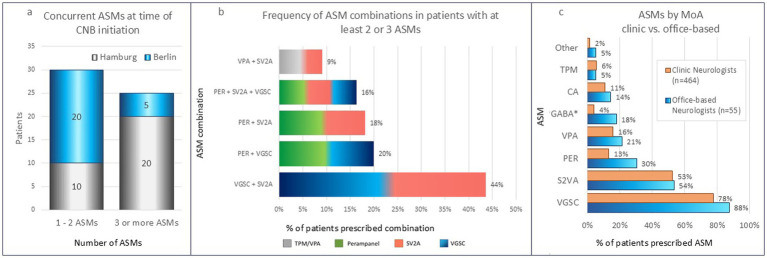
ASM use for the office-based outpatient cohort prior to CNB initiation (n=55). **(a)** 54% patients were taking either 1 or 2 ASMs concurrently, the remainder patients were taking 3 or more ASMs concurrently; **(b)** the most common prior combinations for patients with a minimum 2 concurrent ASMs were VGSC + SV2A blockers (44%), PER + VGSC (20%), PER + SV2A (18%), and PER + SV2A + VGSC (16%). The remaining combinations were given to <10% patients; **(c)** when compared with clinic-based patients and IQVIA™ nationwide population data, the office-based outpatient cohort had a higher prescription of VGSCs, PER, and GABA modulators. These patterns may reflect distinct office-based outpatient treatment strategies. ASM, anti-seizure medication; CNB, Cenobamate; MoA, mechanism of action; n, number of patients; VPA, Valproate; TPM, Topiramate; CA, calcium channel modulator; GABA*, γ-Aminobutyric acid (Clobazam, Primidone/PB, Clonazepam); PER, Perampanel; SV2A, synaptic vesicle protein 2A; VGSC, voltage-gated sodium channel.

The variation in the use of concurrent ASMs prior to CNB start was determined (Figure 3a). The proportion of patients were taking either 1 or 2 ASMs concurrently was 54, and 45% of patients were taking 3 or more ASMs concurrently.

The ASMs prescribed to the study patients was analyzed and the results were as follows: Perampanel (PER) was prescribed to 30% of the patients; voltage-gated sodium channel (VGSC) blockers including LTG (29%) and LCM (25%); synaptic vesicle protein 2A (SV2A) blockers LEV (29%) and Brivaracetam (BRV) (25%); valproate 21% and Oxcarbazepine (OCB) 16% of patients. Smaller numbers of patients were found to be taking various other VGSC blockers, calcium channel modulators, and GABA modulators, these are detailed in Supplementary Table S2.

#### 3.3.2 Prior ASMs by combinations

The most common prior ASM combinations prescribed to patients with a minimum of 2 concurrent ASMs were identified as follows: VGSC + SV2A blockers were prescribed to 43% of patients, PER + VGSC (20%), PER + SV2A (18%), and PER + SV2A + VGSC (16%). The remaining combinations were given to <10% patients (Figure 3b).

#### 3.3.3 Prior ASMs by physician speciality

Data from this study were compared with a larger 2020 cohort (4) of clinic-based patients and nationwide population data from a recent IQVIA™ study examining epilepsy prescription patterns from 2018 to 2022: the German IQVIA™ Disease Analyzer (DA) and Longitudinal Prescriptions (LRx) databases, described in (11, 12) and summarized in Figure 3c. The analysis showed that the largest disparity is seen with PER and GABA followed by VGSC blockers whereas S2VA prescription was consistent between settings.

### 3.4 Qualitative clinician interview

#### 3.4.1 Titration of CNB

Both clinicians reported that the titration of CNB could be easily configured into a quarterly schedule. The advantages of such a slow titration are that most side effects are mitigated and investigations such as plasma measurements are minimized, making the process simpler and less invasive. Detailed titration doses are given in Supplementary Table S1.

#### 3.4.2 ASM combinations for best outcomes after CNB initiation

Table 1 outlines the results from clinician interviews and their subsequent recommendations, including practical tips for individualized titration. Both neurologists reported that prior use of LEV/BRV (SV2A inhibitors) and PER provided combinations with better seizure reduction outcomes than VGSC. Depending on the prior ASM combination, both also recommended reducing the prior ASM when between 50 mg and 150 mg CNB dosage was reached. Observed side effects included sleepiness, fatigue, and dizziness for LTG and LCM as prior ASMs, and insomnia for PER as a prior ASM. For further details, please see Supplementary Table S1.

**Table 1:** Results from clinician interviews and their subsequent recommendations.

Add on to CNB	When adding CNB consider	Side effect most often observed if original ASM reduced too slowly
Levetiracetam (LEV)	No changes necessary.	None reported.
Brivaracetem (BRV)	Few changes required; MoA also includes VGSC.	Sleepiness, fatigue, dizziness.
Lamotrigine (LTG)	After adding CNB, levels of LTG will drop. Reduce gradually as CNB is increased.	Sleepiness, fatigue, dizziness.
Lacosamide (LCM)	Reduce gradually as CNB is increased. Start reduction earlier when LCM is higher dosed.	Sleepiness, fatigue, dizziness.
Perampanel (PER)	After adding CNB, PER levels will drop; if necessary, slightly increase dose. Then, to prevent fatigue and daytime sleepiness, reduce dose gradually as CNB is increased.	Insomnia. Later on: daytime sleepiness, fatigue, dizziness.

Practical tips for individualized CNB titration	Justification
Check LDL levels and add statins, if necessary	Careful monitoring of lipid values can reduce the risk of cardiovascular disease (45). There is some evidence suggesting a protective effect against SUDEP for users of statins (46).
Prescribe vitamin D	Bone health can be negatively affected by CYP 3A4-inducing ASMs (47).
Check contraception for women under 55	Oral contraceptives become ineffective with CYP 3A4-inducing ASMs (48). Switch to hormonal coil is recommended.
Check NOACs	Enzyme-inducing or inhibiting ASMs reduce the effectiveness of anticoagulation produced by NOACs (49–51), especially rivaroxaban + apixaban.

CNB, Cenobamate; ASM, anti-seizure medication; VGSC, voltage-gated sodium channel; MoA, mechanism of action; LDL, low-density lipoprotein cholesterol; SUDEP, sudden unexpected death in epilepsy; CYP 3A4, cytochrome P450 3A4 antibody; NOACs, non-vitamin K antagonist oral anticoagulant agents.

## 4 Discussion

Since its approval in June 2021, Cenobamate has shown unprecedented efficacy as an adjunctive therapy in controlling epileptic seizures (12, 24–32). However, its use by office-based outpatient neurologists in Germany remains relatively limited. By combining physician experiences and data from 55 patients in two office-based German outpatient neurology practices, a picture of office-based perspectives and CNB titration paradigms is provided.

### 4.1 Office-based patient profiles present opportunity for more effective CNB prescription

A comprehensive analysis of the office-based outpatients’ medication histories revealed diverse patterns in the utilization of prior ASMs at the initiation of CNB therapy. VPA, BRV, LEV, and VGSC blockers LTG and LCM all had substantial representation with approximately one quarter of patients using at least one of these medications. Concurrent medication use was also varied. Notably, around half of the patients were taking three or more ASMs and the mean number of failed ASMs was 7.4. These findings underscore that the office-based recipients of CNB were those with refractory epilepsy. With over half of the cohort experiencing improved seizure reduction and concurrent drug load reduction after CNB initiation, there are clear benefits to prescribing CNB to office-based patients with refractory epilepsy.

### 4.2 Improved patient pathways could optimize the role of office-based neurologists

Office-based outpatient neurologists could be instrumental in selecting effective ASM combinations and managing ongoing treatment for their refractory epilepsy patients. Groth et al. postulate that individual patient journeys—from diagnosis to long-term treatment—could potentially influence novel ASM prescription patterns (34). Therefore, dialogues between clinic-based neurologists (who often have first experience with new ASMs) and office-based outpatient neurologists are critical (13). Yet, despite the potential for effective dialogue to improve patient care, data show that only 1% of neurologists refer their epilepsy patients to a specialized treatment center (11, 12), suggesting a need for improved communication and also better access to alternative information sources for ASM prescribing. When an office-based neurologist receives a hospital referral for a patient who has been prescribed a new, unfamiliar ASM, there may be a gap in practical knowledge for optimal titration. This in part explains why a 2023 study reveals that many office-based neurologists perceive CNB as an ASM of last resort (35). However, in our cohort, we showed that two office-based outpatient practices were able to titrate CNB according to published schedules, with positive seizure rate outcomes. Furthermore, drug load was also reduced.

Acute care pathways are also important in the office-based outpatient setting. Office-based physicians should be confident in selecting the optimum ASM prescription when there is an urgent need for work up and prompt, effective ASM therapy because these patients present in the office-based outpatient setting as well as hospital acute care (36–38). If these patients have failed two ASMs, CNB—which has been shown to be highly effective if provided earlier in the disease course (39)—should be considered even in the office-based outpatient setting, as demonstrated by the successful titration and positive outcomes in our cohort.

### 4.3 Comparison of data with reference data

Comparative analysis with clinic-based patient data (4, 11, 12) revealed many similarities in ASM utilization but also some differences. Office-based outpatient neurologists had a higher use of VGSCs, PER, and GABA modulators. These patterns may reflect distinct office-based outpatient treatment strategies such as tailoring prescriptions to individual patient profiles, preference for well-tolerated ASMs, adherence to guidelines, and titration logistics. These differences could also be considered demonstrative of the need for office-based physicians to have data on CNB prescription from office-based patients and not hospital clinic-based patients.

### 4.4 Office-based titration schedules, compliance, and patient satisfaction

In recent years, German and global ASM prescription patterns have shifted toward the use of newer and safer medications, (i.e., second- and third-generation ASMs, including CNB). Hochbaum et al. (4) evaluated epilepsy treatment trends in Germany based on data from four studies conducted between 2008 and 2020. They reported that, in 2020, more than three-quarters of prescribed ASMs in Germany were new-generation medications such as LTG, LEV, and LCM, which aligns with trends observed in other studies in Europe, China, and Japan.

In Germany, these newer ASMs represent the current ASMs of choice, having displaced VPA and Carbamazepine over the last decade. Their increased use reflects a natural response to their ease of titration, more tolerable side effects, good response rates, and lower safety concerns (2). With its recently demonstrated tolerability, CNB also fits well as an ASM choice amongst this group. Clinician reports from this study indicate that CNB could be easily titrated with low side effects. These results are in-line with another real-world evidence cohort (22) studying CNB initiation in highly drug-resistant epilepsy patients in Spain. This Spanish study additionally assessed patient satisfaction, with CNB initiation achieving good scores. Although our study did not include patient satisfaction measures, at the time of initial data collection (June 2023) all patients had full compliance. Indeed, from a patient perspective, there are many benefits to switching to newer ASMs (40, 41), and office-based CNB prescription in Germany can support this. Another Spanish study (42) explored the management of concurrent ASMs along with CNB and concluded in-line with our physicians toward the reduction of high-dosage concurrent ASMs. Therefore, for patients who are currently not seizure-free and being treated with multiple or older ASMs out of alignment with German epilepsy guidelines, CNB can be a good option for reduction of both drug load and side effects (43, 44).

### 4.5 Strengths, limitations, and further research

The focal strength of this preliminary study is that it is the first analysis of CNB use in office-based outpatient settings in Germany, both in terms of which ASMs are being used prior to CNB initiation and which are most amenable in combination with CNB for the improvement of seizure outcomes and reduction of drug load. The data indicate positive clinical outcomes and ease of titration. However, this was a small, first cohort including only two clinicians and two study centers. Future research should expand the data with higher patient numbers and more centers. Another focus for future research is patients with milder epilepsy. This study focused on drug-refractory patients, therefore additional data would need to be collected so that future cohorts can track outcomes for all patient groups. Furthermore, since data collection started just 1 month after the start of CNB titration, long-term outcomes are not yet known. However, following the analysis of these first 55 patients, the involved clinics have continued data collection to about double the original number, so that future papers could provide a cohort update. With regards to outcomes for the reduction of concurrent ASMs and therefore drug load, the scope of this study did not allow for in-depth analysis of these data, however future studies could focus on this, exploring prescription timing and MoA stratifications.

Overall, the unique aspects of office-based outpatient epilepsy practice in Germany are highlighted, showing the different needs and drivers in comparison to hospital-based or specialist outpatient clinics. To address these differences, there is potential for development of an office-based titration scheme. Additionally, further analysis is warranted to explore the statistical significance of the comparison findings and their implications for optimizing epilepsy management.

## 5 Conclusion

Using a first cohort of 55 epilepsy patients, this preliminary study has presented first findings of real-world data on routine CNB titration by office-based outpatient neurologists, identified current ASM prescription combinations for office-based outpatients prior to being prescribed CNB, and compiled a clinicians’ narrative and experience of CNB prescribing. By examining the challenges in epilepsy care pathways throughout Germany, the role of office-based neurologists in refractory epilepsy care is clarified, and the possibility of prescribing CNB in an office-based outpatient setting for improvement of seizure outcomes and drug load reduction is explored. There is a need for prompt, continuing, effective therapy with new-generation ASMs, such as CNB, across all neurological settings in Germany, but there is an especial potential benefit in the office-based outpatient setting.

## IQVIA™ data statement

The data used in this study was collected and evaluated at patient level; nevertheless, this had previously been satisfactorily anonymised in line with German data privacy legislation. Throughout this document, whenever terms such as “patient, doctor, medical practice, prescriber or pharmacy” are used, these therefore do not refer to any personal data but exclusively to anonymous information (in accordance with § 3 Sect. 6 “Bundesdatenschutzgesetz” of the German Federal Data Protection Act).

## Data Availability

The raw data supporting the conclusions of this article will be made available by the authors, without undue reservation.
