# Supplementary material for: Real-world utilization of Cenobamate as adjunct therapy in office-based neurology: practical tips and insights for titration
Source: Front Neurol. 2025 Jun 13;16:1558614. doi: 10.3389/fneur.2025.1558614 (PMC12202365; doi:10.3389/fneur.2025.1558614)
Supplement: Supplementary file 1 [file Table_1.docx]

**Supplemental Information**

Supplemental Table S1

Patient demographics, ASM doses, and seizure outcomes

Please refer to Excel document PDF

Supplemental Table S2

ASM use prior to CNB initiation by MoA, name, and number and percent of patients. Current office-based study compared with reference data (1).

|  |  | 55 office-based patients | | | 464 reference data patients | | |
| --- | --- | --- | --- | --- | --- | --- | --- |
| MoA | Common Name | n patients | % taking ASM | % ASM use | n patients | % taking ASM | % ASM use |
| VPA | Valproate | 12 | 21% | 9% | 74 | 16% | 9% |
| TPM | Topiramate | 3 | 5% | 2% | 26 | 6% | 3% |
| SV2A | Levetiracetam | 16 | 29% | 12% | 154 | 33% | 18% |
| SV2A | Brivaracetam | 14 | 25% | 11% | 90 | 19% | 11% |
| VGSC | Oxcarbazepine | 9 | 16% | 7% | 36 | 8% | 4% |
| VGSC | Eslicarbazepine | 5 | 9% | 4% | 7 | 2% | 1% |
| VGSC | Lacosamide | 14 | 25% | 11% | 96 | 21% | 11% |
| VGSC | Lamotrigine | 16 | 29% | 12% | 194 | 42% | 23% |
| VGSC | Carbamazepine | 3 | 5% | 2% | 26 | 6% | 3% |
| VGSC | Rufinamide | 1 | 2% | 1% | 2 | 0% | 0% |
| VGSC | Phenytoin | 1 | 2% | 1% | 1 | 0% | 0% |
| PER | Perampanel | 17 | 30% | 13% | 61 | 13% | 7% |
| CA | Pregabalin | 3 | 5% | 2% | 10 | 2% | 1% |
| CA | Zonisamide | 3 | 5% | 2% | 35 | 8% | 4% |
| CA | Ethosuximide | 1 | 2% | 1% | 5 | 1% | 1% |
| CA | Methsuximide | 1 | 2% | 1% | 1 | 0% | 0% |
| GABA | Clobazam | 4 | 7% | 3% | 0 | 0% | 0% |
| GABA | Primidone/PB | 4 | 7% | 3% | 12 | 3% | 1% |
| GABA | Clonazepam | 2 | 4% | 2% | 7 | 2% | 1% |
| OTH | Stiripentol | 1 | 2% | 1% | 0 | 0% | 0% |
| OTH | Fenfluramine | 1 | 2% | 1% | 1 | 0% | 0% |
| OTH | Cannabidiol | 1 | 2% | 1% | 5 | 1% | 1% |
| OTH | Gabapentin | 0 | 0% | 0% | 3 | 0% | 0% |
| CNB | Cenobamate | 55 | 100% | 100% | 0 | 0% | 0% |

Abbreviations: n, number of patients; MoA, mechanism of action; VPA, Valproate; TPM, Topiramate; d; GABA, γ-Aminobutyric acid; PER, Perampanel; SV2A, synaptic vesicle protein 2A; VGSC, voltage-gated sodium channel; OTH, other.

References

1. Hochbaum M, Kienitz R, Rosenow F, Schulz J, Habermehl L, Langenbruch L, et al. Trends in antiseizure medication prescription patterns among all adults, women, and older adults with epilepsy: A German longitudinal analysis from 2008 to 2020. Epilepsy Behav. 2022 May;130:108666.
